# Potential diagnostic value of CSF metabolism-related proteins across the Alzheimer’s disease continuum

**DOI:** 10.1186/s13195-023-01269-8

**Published:** 2023-07-15

**Authors:** Silvia Paciotti, Anna Lidia Wojdała, Giovanni Bellomo, Andrea Toja, Elena Chipi, Sander R. Piersma, Thang V. Pham, Lorenzo Gaetani, Connie R. Jimenez, Lucilla Parnetti, Davide Chiasserini

**Affiliations:** 1 Section of Physiology and Biochemistry, Department of Medicine and Surgery, University of Perugia, Perugia, Italy; 2 Laboratory of Clinical Neurochemistry, Department of Medicine and Surgery, University of Perugia, Perugia, Italy; 3 Section of Neurology, Department of Medicine and Surgery, University of Perugia, Perugia, Italy; 4 OncoProteomics Laboratory, Laboratory Medical Oncology, Amsterdam University Medical Center, Amsterdam, The Netherlands

**Keywords:** Alzheimer’s disease, Cerebrospinal fluid, Biomarkers, Preclinical AD, Pyruvate kinase, Aldolase, Ubiquitin C-terminal hydrolase L1, Fatty acid-binding protein 3

## Abstract

**Background:**

Alzheimer’s disease (AD) cerebrospinal fluid (CSF) core biomarkers (Aβ42/40 ratio, p-tau, and t-tau) provide high diagnostic accuracy, even at the earliest stage of disease. However, these markers do not fully reflect the complex AD pathophysiology. Recent large scale CSF proteomic studies revealed several new AD candidate biomarkers related to metabolic pathways. In this study we measured the CSF levels of four metabolism-related proteins not directly linked to amyloid- and tau-pathways (i.e., pyruvate kinase, PKM; aldolase, ALDO; ubiquitin C-terminal hydrolase L1, UCHL1, and fatty acid-binding protein 3, FABP3) across the AD continuum. We aimed at validating the potential value of these proteins as new CSF biomarkers for AD and their possible involvement in AD pathogenesis, with specific interest on the preclinical phase of the disease.

**Methods:**

CSF PKM and ALDO activities were measured with specific enzyme assays while UCHL1 and FABP3 levels were measured with immunoassays in a cohort of patients composed as follows: preclinical AD (pre-AD, *n *= 19, cognitively unimpaired), mild cognitive impairment due to AD (MCI-AD, *n* = 50), dementia due to AD (ADdem, *n* = 45), and patients with frontotemporal dementia (FTD, *n* = 37). Individuals with MCI not due to AD (MCI, *n* = 30) and subjective cognitive decline (SCD, *n* = 52) with negative CSF AD-profile, were enrolled as control groups.

**Results:**

CSF UCHL1 and FABP3 levels, and PKM activity were significantly increased in AD patients, already at the pre-clinical stage. CSF PKM activity was also increased in FTD patients compared with control groups, being similar between AD and FTD patients. No difference was found in ALDO activity among the groups.

UCHL1 showed good performance in discriminating early AD patients (pre-AD and MCI-AD) from controls (AUC ~ 0.83), as assessed by ROC analysis. Similar results were obtained for FABP3. Conversely, PKM provided the best performance when comparing FTD vs. MCI (AUC = 0.80). Combination of PKM, FABP3, and UCHL1 improved the diagnostic accuracy for the detection of patients within the AD continuum when compared with single biomarkers.

**Conclusions:**

Our study confirmed the potential role of UCHL1 and FABP3 as neurodegenerative biomarkers for AD. Furthermore, our results validated the increase of PKM activity in CSF of AD patients, already at the preclinical phase of the disease. Increased PKM activity was observed also in FTD patients, possibly underlining similar alterations in energy metabolism in AD and FTD.

**Supplementary Information:**

`The online version contains supplementary material available at 10.1186/s13195-023-01269-8.

## Background

Alzheimer’s disease (AD) is a neurodegenerative disorder associated with progressive cognitive, behavioural, and functional impairment. AD progresses along a continuum, that ranges from an extended preclinical disease (asymptomatic phase), through mild cognitive impairment (MCI) to the final stage of dementia [1].

The preclinical phase begins 10 to 20 years before the onset of clinical symptoms [1]. During this initial phase, neurodegeneration has already started, with a gradual loss of synapses and neurons, possibly caused by extracellular accumulation of amyloid-β (Aβ) plaques and formation of intracellular neurofibrillary tangles of hyperphosphorylated tau protein (p-tau) [2, 3].

Currently, the diagnosis of AD is based on the combination of clinical evaluation and neuropsychological assessment together with brain imaging and cerebrospinal fluid (CSF) biomarkers. Screening tools able to identify AD patients at the earliest stages of the disease have become of fundamental importance in clinical trials, where disease-modifying therapies may have better chances of success if implemented before the occurrence of an overt neurodegeneration.

Within this paradigm, the 2018 NIA-AA (National Institute on Aging – Alzheimer's Association) research framework [1] proposed to shift the definition of AD from a clinical-pathological construct towards a biological entity, characterized by the association of AD-related neuropathological processes with changes in biological markers. The core biomarkers are integrated within the AT(N) system, an open categorical classification procedure that considers Amyloidosis, Tauopathy, and Neurodegeneration biomarkers [4]. The concomitant presence of amyloidosis and tauopathy biomarkers is mandatory for the biomarker-based diagnosis of AD, while neurodegeneration markers, which are not specific for AD, are rather used to stage disease severity. Importantly, the research framework combines the cognitive staging of AD across the entire spectrum of the disease (i.e., cognitively unimpaired, MCI, and dementia phase) with the biomarker characterization via the AT(N) system. The cognitively unimpaired subjects with biomarker positivity for amyloidosis and tauopathy (A + /T +) are classified as preclinical AD (pre-AD). This approach formally defines this group of AD patients exhibiting molecular alterations before the onset of cognitive deficits and is therefore of utmost interest for investigating new candidate biomarkers or the pathological processes of the early disease.

The measurement of amyloidosis and tauopathy biomarkers in CSF (i.e., Aβ42/40 ratio, p-tau, and total tau (t-tau)) provides high diagnostic accuracy, even at the preclinical stage of AD [5]. However, these biomarkers do not fully describe the complex and multifactorial pathogenic process taking place in AD across its different stages. Indeed, even though the dysregulation of the amyloid processing and tau-related hyperphosphorylation and neurodegeneration are at the core of the biological definition of AD, several other molecular changes have been reported across the Alzheimer’s continuum [6]. Furthermore, the limited results of amyloid-targeting therapies demonstrate that additional pathological pathways are implicated in neurodegeneration leading to AD dementia [7]. Accordingly, the use of global and unbiased proteomic approaches has been instrumental for identification of new AD biomarkers, linked to pathways different from those currently assessed [8–12]. For instance, recent studies integrated mass spectrometry data across multiple cohorts to obtain consensus modules and clusters of proteins showing altered levels in CSF and brain of AD patients [8–11]. Among the pathways of interest, energy metabolism, immune response but also lipid metabolism and protein processing have shown consistent alteration in CSF of AD patients [8, 10, 11]. However, some of these candidate biomarkers have never been validated in large cohorts including AD patients at different stages. In this work, we focused on measuring the activity of pyruvate kinase isoform M (PKM) and aldolase (ALDO) two glycolytic enzymes that have been consistently found increased in AD CSF [13], together with the levels of ubiquitin C-terminal hydrolase L1 (UCH-L1), and fatty acid-binding protein 3 (FABP3), two proteins involved respectively in protein processing [14] and lipid metabolism [15], also found to be increased in AD CSF in large scale proteomic studies [9, 10].

The four candidates were measured here in a large and well-characterized cohort including patients affected by AD at different stages (i.e., preclinical AD, MCI due to AD, and AD dementia). The patients within the AD continuum were compared with control groups (MCI not due to AD and asymptomatic individuals with subjective cognitive decline) and with patients affected by frontotemporal dementia (FTD), representing the second most prevalent neurodegenerative dementia. We aimed at evaluating the potential role of PKM, ALDO, FABP3, and UCHL1 as CSF biomarkers useful to characterise the AD continuum and understand the role of metabolic pathways in early AD pathogenesis [16].

## Methods

### Study participants

The patients included in this study were enrolled at the Centre of Memory Disturbances of the University of Perugia. The cohort included 233 patients evaluated in our center between 2013 and 2021. All patients underwent a standardized assessment including medical history, physical and neurological examination, laboratory tests, and neuropsychological evaluation including Mini-Mental State Examination (MMSE). To obtain a complete characterization of early AD patients (pre-AD and MCI-AD), a comprehensive neuropsychological battery was used. It included traditional paper–pencil tests, such as Trail Making test (TMT), Digit Symbol Substitution Test (DSST), Rey- Auditory Verbal Learning Test (RAVLT), and, in a small subset (*n* = 41) also computerized tasks from Cambridge Neuropsychological Test Automated Battery (CANTAB). Brain imaging (computed tomography or magnetic resonance imaging, MRI) or ^18^Fluoro-2-deoxyglucose positron emission tomography (FDG-PET), were also performed in selected cases, according to clinical suspicion. Demographic and clinical data of all the patients included in the study are reported in Table 1. All the patients undergone CSF analysis using automated Lumipulse® technology for Aβ42/Aβ40 ratio, t-tau, and p-tau 181. For patient initially screened with other non-automated immunoassays (2013 -2018) the measurements were repeated with the Lumipulse® platform. The patients were then classified as A + /A-, T + /T-, N + /N- according to the method described by Bellomo et al. [17] (cut-off values Aβ42/Aβ40 = 0.072, 95% CI 0.07–0.074; t-tau = 50, 95% CI 46.2–52.3; *p*-tau 181 = 393, 95% CI 359–396).Briefly, after assessing the proportion of AD cases and control cases on clustered data, cut-off values were assessed by reclassifying the whole cohort, significantly increasing the sample size [17]. The cohort enrolled in this work was composed of AD patients at different stages: 45 patients affected by dementia due to AD (ADdem), 50 patients with mild cognitive impairments due to AD (MCI-AD), and 19 cognitively unimpaired subjects with preclinical Alzheimer’s disease (pre-AD), all diagnosed according to the 2018 NIA-AA criteria with a A + /T + CSF profile [1]. We also included 30 patients affected by mild cognitive impairments not due to AD (MCI) [18] and 37 patients affected by FTD [19, 20], none of them having A + /T + CSF profile. As a control group, we selected 52 subjects with subjective cognitive decline (SCD) [21] who underwent CSF analysis for diagnostic reasons. SCD group included both subjects with a normal CSF profile or a non-AD biomarkers profile showing tauopathy and/or neurodegenerative biomarkers, as well as 3 subjects with amyloidosis. SCD individuals with the concurrent presence of amyloidosis and tauopathy (A + /T +) were excluded from the study. Table 1 reports the AT(N) classification for all the patients included in this study, grouped for diagnosis. For analysing the correlations with cognitive scores, we considered patients with a follow-up of at least 1.5 years (*n* = 77, range 1.5–8 years).

**Table 1:** Demographics and clinical features of the selected cohort

Variable	Overall	SCD	MCI	pre-AD	MCI-AD	ADdem	FTD
**n**	233	52	30	19	50	45	37
Age (years) mean ± SD	70.5 ± 7.8	65.5 ± 9.0	71.0 ± 8.1	73.6 ± 5.8	72.5 ± 5.9	74.0 ± 6.4	68.4 ± 6.4
Sex (male) n (%)	99 (42.5)	28 (53.8)	14 (46.7)	6 (31.6)	16 (32.0)	15 (33.3)	20 (54.1)
MMSE median [IQR]	25.0 [22.0, 28.0]	28.0 [27.8, 29.0]	25.0 [23.2, 28.0]	27.0 [27.0, 28.5]	24.0 [22.0, 27.0]	15.0 [11.0, 19.0]	25.0 [23.0, 26.0]
Years of Education mean ± SD	9.4 ± 4.6	10.9 ± 4.3	9.0 ± 5.0	12.2 ± 3.5	9.1 ± 4.8	7.6 ± 3.8	9.5 ± 4.5
**ATN profile, n (%)**							
A-T-N-	73 (31.3)	38 (73.1)	17 (56.7)	0 (0.0)	0 (0.0)	0 (0.0)	18 (48.6)
A + T-N-	14 (6.0)	3 (5.8)	4 (13.3)	0 (0.0)	0 (0.0)	1 (2.2)	6 (16.2)
A + T + N-	14 (6.0)	0 (0.0)	0 (0.0)	7 (36.9)	7 (14.0)	0 (0.0)	0 (0.0)
A + T + N +	96 (41.2)	0 (0.0)	0 (0.0)	12 (63.2)	42 (84.0)	44 (97.8)	0 (0.0)
A-T-N +	5 (2.1)	1 (1.9)	2 (6.7)	0 (0.0)	0 (0.0)	0 (0.0)	2 (5.4)
A-T + N-	15 (6.4)	6 (11.5)	5 (16.7)	0 (0.0)	0 (0.0)	0 (0.0)	4 (10.8)
A-T + N +	14 (7.0)	4 (7.7)	2 (6.7)	0 (0.0)	1 (2.0)	0 (0.0)	7 (18.9)
**AD profile, n (%)**							
Normal AD biomarkers	73 (31.3)	38 (73.1)	17 (56.7)	0 (0.0)	0 (0.0)	0 (0.0)	18 (48.6)
AD pathologic change	14 (6.0)	3 (5.8)	4 (13.3)	0 (0.0)	0 (0.0)	1 (2.2)	6 (16.2)
AD profile	112 (48.0)	0 (0.0)	0 (0.0)	19 (100.0)	49 (98.0)	44 (97.8)	0 (0.0)
Non-AD pathologic change	34 (15.6)	11 (21.2)	9 (30.0)	0 (0.0)	1 (2.0)	0 (0.0)	13 (35.1)

*SCD* Subjective cognitive decline *MCI* Mild cognitive impairment *pre-AD* preclinical Alzheimer’s disease *MCI-AD* Mild cognitive impairment due to Alzheimer’s disease *ADdem* Alzheimer’s disease with dementia, *FTD* Frontotemporal dementia *IQR* Interquartile range *MMSE* Mini-Mental State Examination *SD* Standard deviation

All the procedures involving human subjects were performed following Helsinki Declaration. All the patients and/or their legal representatives gave informed written consent for the lumbar puncture and the inclusion in the study that was approved by the local Ethics Committee (Comitato Etico Aziende Sanitarie Regione Umbria 19,369/AV and 20,942/21/OV).

### Human CSF samples

Lumbar puncture was performed according to international guidelines [22–24] and standardized procedures [25]. Briefly, 10–12 mL of CSF was collected in sterile polypropylene tubes (Sarstedt 62.610.210) and centrifuged for 10 min (2000 × g), at room temperature. Aliquots of 0.5 mL were frozen at -80 °C in polypropylene tubes (Sarstedt 72.730.007) pending analysis.

The levels of the CSF AD biomarkers (Aβ1-42/Aβ40 ratio, t-tau, and p-tau) were measured in all the samples by using the Lumipulse G600-II platform (Fujirebio Inc).

### Development of colorimetric assays for evaluating CSF ALDO and PKM activities

CSF ALDO and PKM activities were assessed by establishing two colorimetric NADH-coupled reaction assays starting from published protocols [26, 27]. Briefly, PKM activity was measured by incubating 100 µL of CSF with 200 µL of reaction buffer composed of 1 M MgCl_2_, 2.5 M KCl, 0.1 M ADP, 0.013 M NADH, 0.155 M PEP, and 7 U/mL of LDH in 0.2 M Imidazole–HCl buffer, pH 7.2. Similarly, CSF ALDO activity was measured by incubating 150 µL of each CSF sample with 150 µL of 87 mM Tris–HCl buffer containing 1.9 mM Fructose 1,6 diphosphate, 0.13 mM NADH, and 1.7 U/mL of α-Glycerophosphate Dehydrogenase/Triosephosphate Isomerase enzyme solution. Our assays for PKM and ALDO measure an enzyme activity relative to all the isoforms possibly present in CSF for both enzymes [28, 29]. The reactions were monitored in a Clariostar (BMG Labtech, Germany) plate reader. For both ALDO and PKM, an enzymatic activity was calculated by recording the absorbance (OD) at 340 nm for 40 min at 25 °C and considering the decrease in absorbance from the initial linear portion of the curve. One unit of ALDO is defined as the amount of enzyme which converts one micromole of fructose 1.6 diphosphate to dihydroxyacetone phosphate and glyceraldehyde 3-phosphate, while one unit of PKM causes the oxidation of one micromole of NADH per minute.

Two pre-constituted CSF pools were loaded in each run as internal quality control. For both assays the intra-run coefficient of variation was < 10% and the inter-run coefficient of variation was < 20% (Supplementary Table 1).

### Measurement of CSF UCHL1 and FABP3 levels

CSF UCHL1 levels were measured using the Human UCHL1/PGP.5 DuoSet ELISA (R&D Systems DY6007-05). Reaction wells of a 96-well plate (Greiner bio-one, code 655,061) were coated with mouse anti-Human UCHL1 capture antibody (150 µg/well) in PBS and incubated overnight (ON) at room temperature (RT). Plates were then emptied out, washed 4 times with a buffer containing 0.05% Tween-20 in PBS and blocked for 2 h at RT with 1% BSA-PBS. The plates were washed 3 times, and Human UCHL1 Standard (ranging from 3.91 pg/well to 250 pg/well) and 100 µL of each CSF samples, were loaded and incubated ON at 4 °C with a constant shaking (450 rpm). After washing, biotinylated sheep anti-Human UCHL1 detection antibody was added to each well (188 µg/well) and the plates were incubated for 2 h at RT (450 rpm). The plates were further washed and incubated with 100 µL/well of Streptavidin-HRP diluted in 1% BSA-PBS for 20 min at RT (450 rpm). After additional washing, the wells were developed with the TMB solution. The reaction was stopped with 100 µL of 1 M H_2_SO_4_. The absorbance (450 nm and 630 nm) was read in a Clariostar (BMG Labtech, Germany) plate reader.

CSF FABP3 levels were measured by using a commercially available kit (Hycult Biotech, HK402) and following previously published procedures [30].

For both UCHL1 and FABP3 measurements, 2 pre-constituted CSF pools were loaded as an internal quality control in each run. For UCHL1, the intra-assay coefficient of variation (CV) was 2.9% while the inter-assay CV was 3.0% (Supplementary Table 1). Limit of detection was 14.8 pg/mL. For FABP3 intra-assay CV was 3.4% while the inter-assay one was 9.7%, the limit of detection corresponded to 102 pg/mL.

### *APOE* genotyping

*APOE* genotype was available for 93 out of 233 patients included in this study (AD, *n* = 18; MCI-AD, *n* = 30; pre-AD, *n* = 9; MCI, *n* = 12; SCD, *n* = 17; FTD, *n* = 7). Genotyping was carried out by using DNA microarrays kit (EUROArray *APOE* Direct, Euroimmun) and following the manufacturer’s instructions.

### Statistical analysis

The data analysis was performed by using the R software v 3.6 [31]. Shapiro–Wilk test was applied to assess data normality distribution. To determine the optimal sample size, a power analysis was performed on previous data collected for FABP3 [32]. According to the results, a minimum of 25–30 patients for the AD and control groups had to be included in the cohort to obtain significant differences in biomarker distributions (*p*-value < 0.05, power t-test ≥ 0.8). Parametric or non-parametric tests were applied when appropriate. Linear regression analyses were performed to assess the effect of storage time in days on biomarker levels. β expresses the slope of the linear regressions in picograms per millilitre per day ± standard error of the mean (SEM).

To assess the statistical significance of the tested biomarkers among the diagnostic groups, analysis of covariance (ANCOVA) on ranked variables was applied, using age, sex, and *APOE* genotype as covariates. Multiple comparison correction was performed using Tukey’s test. Correlations were calculated according to Spearman and Bonferroni correction was used in case of multiple correlations testing. pROC package [33] was used to assess the diagnostic performance of the biomarkers using Receiver Operator Characteristics analysis (ROC). ROC analysis parameters (area under the curve, AUC, 95% confidence intervals, sensitivity, specificity, and cut-off values) were calculated using 2000 bootstrap replicates. Cut-off values were calculated using Youden’s index. Multivariable analysis was performed using stepwise logistic regression (glm function in R, package stats), the stepAIC function from the package MASS [34] was used for model selection according to Akaike Information Criterion. Comparisons among the ROC curves were made using the DeLong test from the function roc.test (package pROC).

## Results

### Effect of the long-term storage on the candidate biomarkers

We evaluated the effect of the long-term storage on the stability of ALDO, PKM, FABP3, and UCHL1 by measuring either the activity or the levels of the candidate biomarkers in CSF samples selected for this study (*n* = 233), which were collected between 2013 and 2021 (Supplementary Fig. 1). Linear regression analysis showed that there was no significant relation between storage time and biomarkers values (PKM, β [slope ± SEM] = 0.0007 ± 0.0004 mEU/mL per day *p* = 0.052; ALDO, β = 0.00009 ± 0.00005 mEU/mL per day *p* = 0.084; UCHL1, β = -0.044 ± 0.023 pg/mL per day *p* = 0.060; FABP3, β = 0.010 ± 0.020 pg/mL per day *p* = 0.576).

### Demographic and clinical data of the selected cohort

Demographic and clinical features of the cohort are reported in Table 1.

A significant difference in terms of age was observed among the groups (*p* < 0.0001). AD (pre-AD, MCI-AD, and ADdem) and MCI patients showed higher mean age compared with SCD group, while FTD patients did not show any difference when compared with MCI and SCD. The frequency of male and female among the groups was similar.

We evaluated the difference in the cognitive performance of the patients considering the MMSE score recorded at the baseline. As expected, it was significantly lower in all AD groups (MCI, MCI-AD, and ADdem) compared with SCD. Considering AD patients, MMSE decreased across pre-AD, MCI-AD, and ADdem groups, with ADdem having the lowest value. FTD showed significantly lower MMSE values compared with SCD subjects and pre-AD patients, but no difference was observed in comparison with MCI and MCI-AD patients (for all the comparisons the results of the statistical analysis are reported in Supplementary Table 2).

### Levels of the CSF candidate biomarkers in the diagnostic groups

The levels and the activity of the candidate biomarkers and the concentration of the core AD biomarkers are reported in Supplementary Table 2, while the patterns of the core CSF AD biomarkers Aβ42/40 ratio, p-tau, and t-tau, used for AT(N) classification, are reported in Supplementary Fig. 2.

**Fig. 1:**
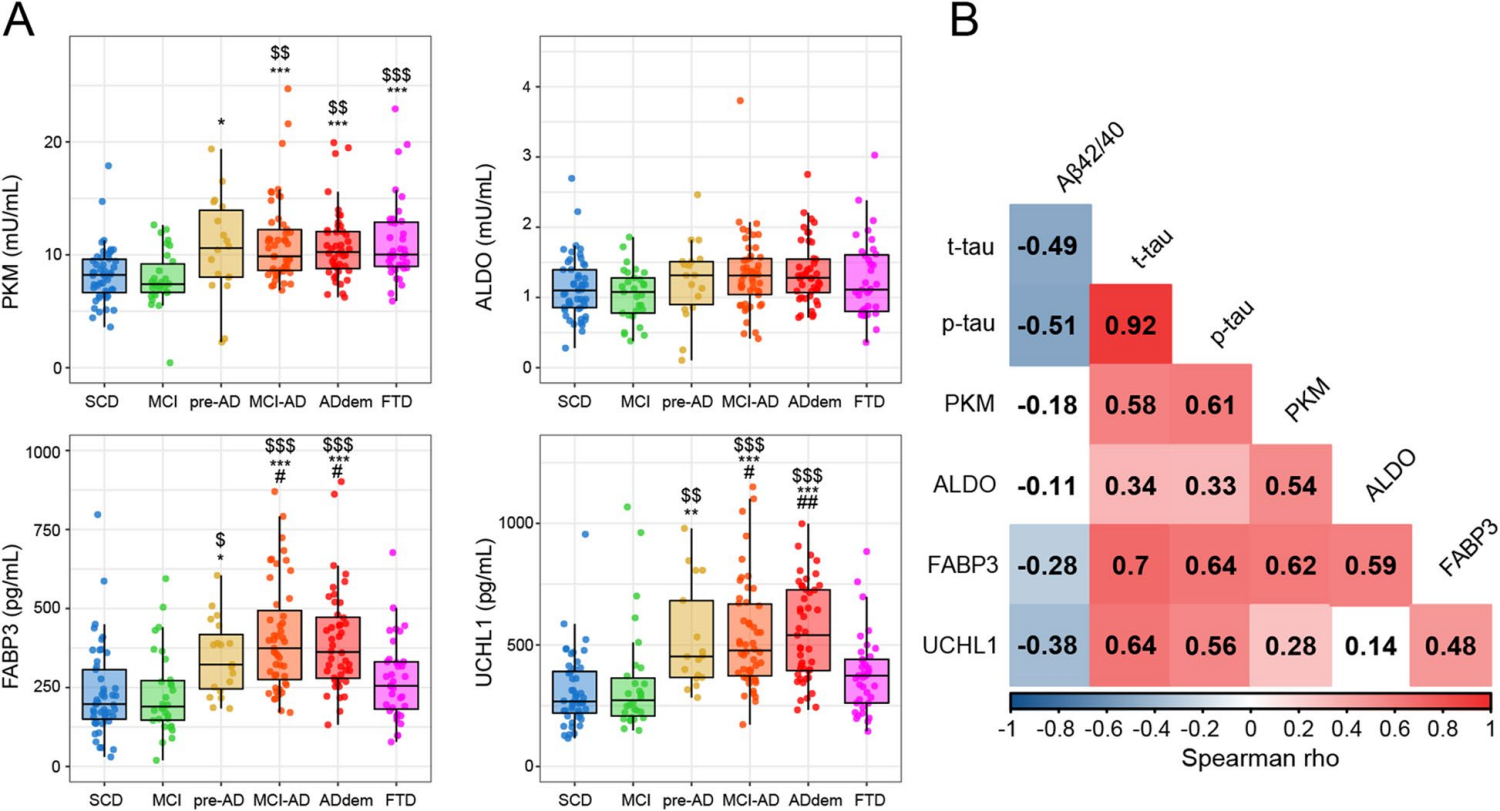
Levels of biomarkers across the diagnostic groups. **A** Combined boxplots and scatter plots showing the CSF activity of PKM (mU/mL) and ALDO (mU/mL) and the concentration of FABP3 (pg/mL) and UCHL1 (pg/mL) in the diagnostic groups presented as median and interquartile range. Analysis was performed by ANCOVA. Symbols indicate the significant comparisons versus SCD ($), MCI (*) and FTD (#). The number of symbols indicates the level of statistical significance: p < 0.05, p < 0.01, p < 0.001. **B** Correlation plot (Spearman) for the CSF biomarkers panel in the whole cohort. White colour indicates no significant correlation

The difference between the biomarkers across the diagnostic groups were evaluated by ANCOVA including age, sex, and *APOE* genotype as covariates. Analysing the new candidate CSF biomarkers, significantly higher PKM activity was found in ADdem and MCI-AD patients compared with SCD and MCI groups. Pre-AD subjects also showed higher PKM activity than the MCI group. In FTD, PKM activity was increased compared to both SCD and MCI. Conversely, no significant difference in ALDO activity was found among the groups (Fig. 1 A and Supplementary Table 1 and Supplementary Table 2).

The concentration of UCHL1 was significantly higher in AD patients (pre-AD, MCI-AD, ADdem) with respect to SCD and MCI. In FTD, UCHL1 levels were not significantly different compared with control groups (Fig. 1 A and Supplementary Table 1 and Supplementary Table 2). FABP3 levels in CSF were significantly higher across the AD continuum compared with SCD and MCI (Fig. 1 A and Supplementary Table 1 and Supplementary Table 2), whereas the levels of FABP3 in FTD patients were similar to those measured in MCI and SCD groups. Interestingly, the levels of both UCHL1 and FABP3 significantly increased already in pre-AD patients compared with SCD and MCI, indicating that these changes are present in cognitively unimpaired patients with a positive CSF profile for the core AD biomarkers.

### Correlation analyses

The correlation analysis among new candidate biomarkers and classical AD CSF biomarkers is reported in Fig. 1B, while Supplementary Table 3 includes the correlation of the candidate biomarkers with the demographic and neuropsychological parameters.

A significant correlation with age was documented for the classical CSF AD biomarkers, namely Aβ42/40 ratio (*r *= -0.40, *p* < 0.001), t-tau (*r* = 0.28, *p* < 0.001), and p-tau (*r* = 0.27, *p* < 0.001), as well as for the candidate biomarkers: UCHL1 (*r* = 0.24, *p* < 0.001), FABP3 (*r* = 0.29, *p* < 0.001), PKM (*r* = 0.29, *p* < 0.001), and ALDO (*r* = 0.26, *p* < 0.001). UCHL1 and FABP3 were significantly associated with the Aβ42/40 ratio (*r* = -0.38, *p* < 0.001; *r* = -0.28, *p* < 0.001). Both t-tau and p-tau exhibited stronger positive correlations with UCHL1 (*r* = 0.64, *p* < 0.001; *r* = 0.56, *p* < 0.001), FABP3 (*r* = 0.70, *p* < 0.001; *r* = 0.64, *p* < 0.001), and PKM (*r* = 0.58, *p* < 0.001; *r* = 0.61, *p* < 0.001). PKM also correlated with ALDO (*r* = 0.54, *p* < 0.001), UCHL1 (*r* = 0.28, *p* < 0.001), and FABP3 (*r* = 0.62, *p* < 0.001). FABP3 correlated with ALDO (*r* = 0.59, *p* < 0.001) and UCHL1 (*r* = 0.48, *p* < 0.001).

We also evaluated the association between the CSF biomarkers and MMSE at baseline (Supplementary Table 3). The strongest correlations were found with the core AD biomarkers. We found a significant correlation between Aβ42/40 ratio, t-tau, and p-tau with MMSE (*r* = -0.30, *r* = -0.41, *r* = -0.33; *p* < 0.001 for all the correlations). For the new candidate biomarkers, after adjusting for multiple comparisons, we found a weak, though significant correlation with MMSE at the baseline for UCHL1 (*r* = -0.27, *p* < 0.001).

We also assessed whether the levels of the candidate biomarkers were associated with the annual decline in MMSE in a subgroup composed of 77 patients followed-up for at least 1.5 years (SCD *n* = 16, MCI *n* = 7, pre-AD *n* = 15, MCI-AD *n* = 22, AD *n* = 6, FTD *n* = 11) No significant correlations were found for any of the biomarkers analysed.

### Diagnostic performance of the new candidate biomarkers

**Fig. 2:**
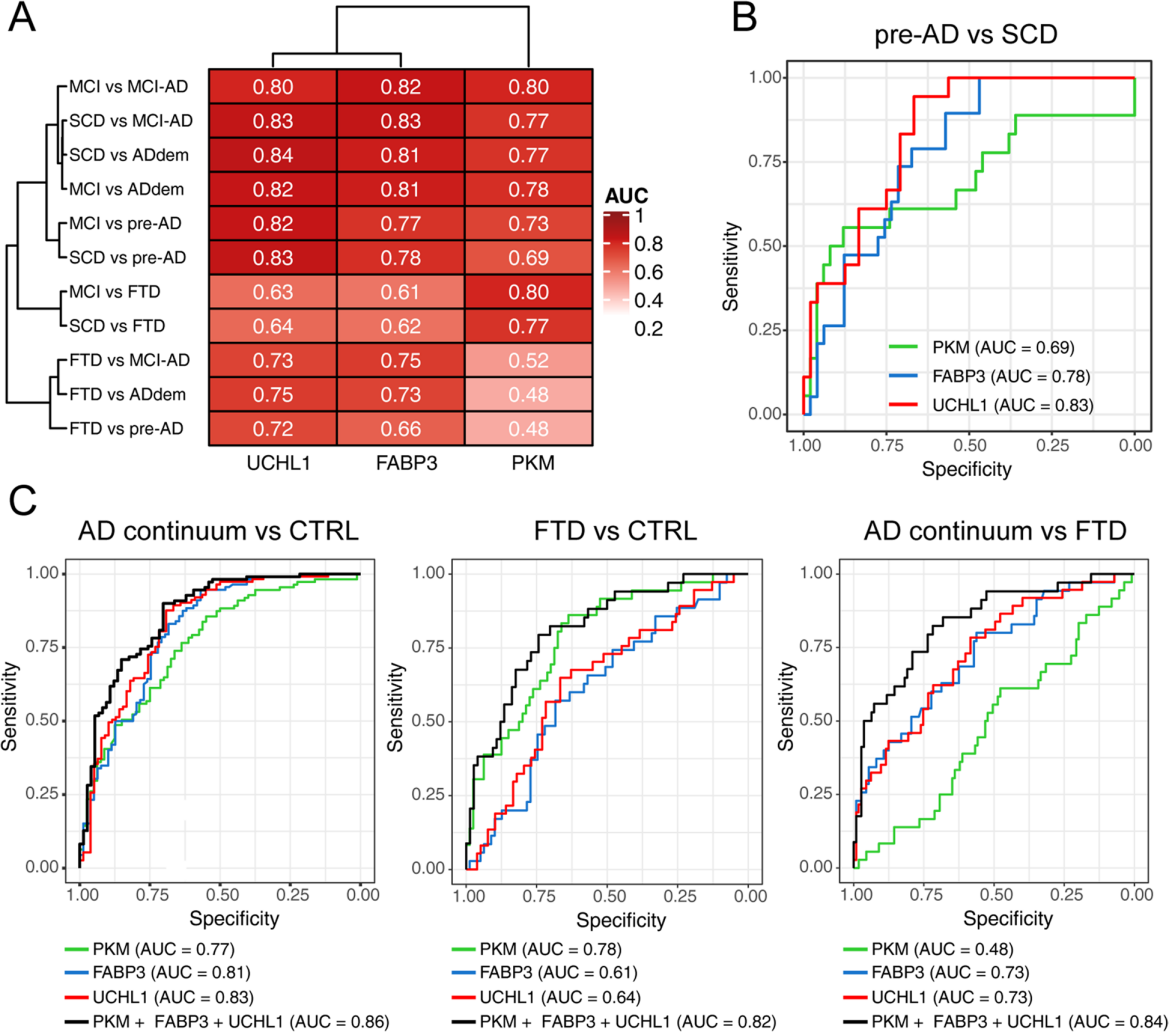
Diagnostic performance of the candidate CSF biomarkers PKM, FABP3, and UCHL1. **A** Heatmap of the AUCs of the single biomarkers for all diagnostic comparisons. **B** ROC curves of the candidate biomarkers in differentiating control subjects (SCD and MCI; CTRL) from AD patients (AD continuum; pre-AD, MCI-AD, and ADdem) and FTD, as well as FTD from AD continuum. Univariate ROC analysis was performed to assess the diagnostic value of the single biomarkers, whereas the diagnostic performance of the combined biomarkers was evaluated using a logistic regression model

The diagnostic performance of the new candidate biomarkers PKM, UCHL1, and FABP3 was first assessed by univariate ROC analysis (the complete analysis for each biomarker, including sensitivity and specificity and confidence intervals is reported in Supplementary Table 4). Since ALDO activity was similar across different diagnostic groups, it was excluded from further analyses. In Fig. 2A we summarized the AUCs for the different markers and comparisons using a clustered heatmap. UCHL1 and FABP3 showed a very similar performance in distinguishing ADdem and MCI-AD patients from SCD and MCI with AUCs ranging from 0.81 to 0.84 (Fig. 2A). For pre-AD patients, UCHL1 was superior to FABP3 and PKM showing an AUC of 0.82 vs. MCI patients and 0.83 vs. SCD (Fig. 2A, B). Conversely FABP3 and PKM accuracy was lower, showing an AUC of 0.78 and 0.69 respectively (pre-AD vs. SCD, Fig. 2B).

When UCHL1 and FABP3 were used for differentiating ADdem from FTD an accuracy of 0.75 and 0.73 was obtained, respectively. Conversely, PKM was not able to achieve an adequate discrimination between ADdem and FTD (AUC 0.48, Fig. 2A), suggesting that similar pathological processes involving the glycolytic pathway take place in patients affected by these two neurodegenerative diseases. On the other hand, PKM provided the best performance for the comparisons FTD vs. SCD (AUC 0.77) and FTD vs. MCI (AUC 0.80), for which UCHL1 and FABP3 showed lower diagnostic accuracy (AUC within the range 0.61–0.64).

The diagnostic groups within the AD continuum (pre-AD, MCI-AD, and ADdem) showed similar levels of UCHL1 and FABP3 and similar activity of PKM. With the aim to understand if, in numerically larger groups, the combination of biomarkers was superior to the single biomarkers, we grouped the pre-AD, MCI-AD, and ADdem in an “AD continuum” group and SCD / MCI in a control group (CTRL). Subsequently, we performed multivariate analysis of the combination vs. the single biomarkers for different comparisons, also against the FTD group. Using logistic regression with stepwise backward model selection, we found that in all the tested comparisons, the three biomarkers were always retained in the final models. For the comparison AD continuum vs. CTRL, the combination of the three biomarkers slightly improved the diagnostic performance when compared to UCHL1 alone (AUC = 0.87, specificity 70% sensitivity 90%), but without reaching statistical significance (*p* = 0.36). For the FTD vs. CTRL and FTD vs. AD continuum comparisons we obtained similar results, with an increase of AUC, sensitivity, and specificity but not significantly different from the best performing biomarkers used in isolation (PKM for FTD vs. CTRL, *p* = 0.59; UCHL1 for FTD vs. AD continuum *p* = 0.09, DeLong test). Globally, the combination of biomarkers somehow showed better diagnostic parameters but without having a clear-cut improvement in performance.

### CSF biomarker levels and *APOE* genotyping

**Fig. 3:**
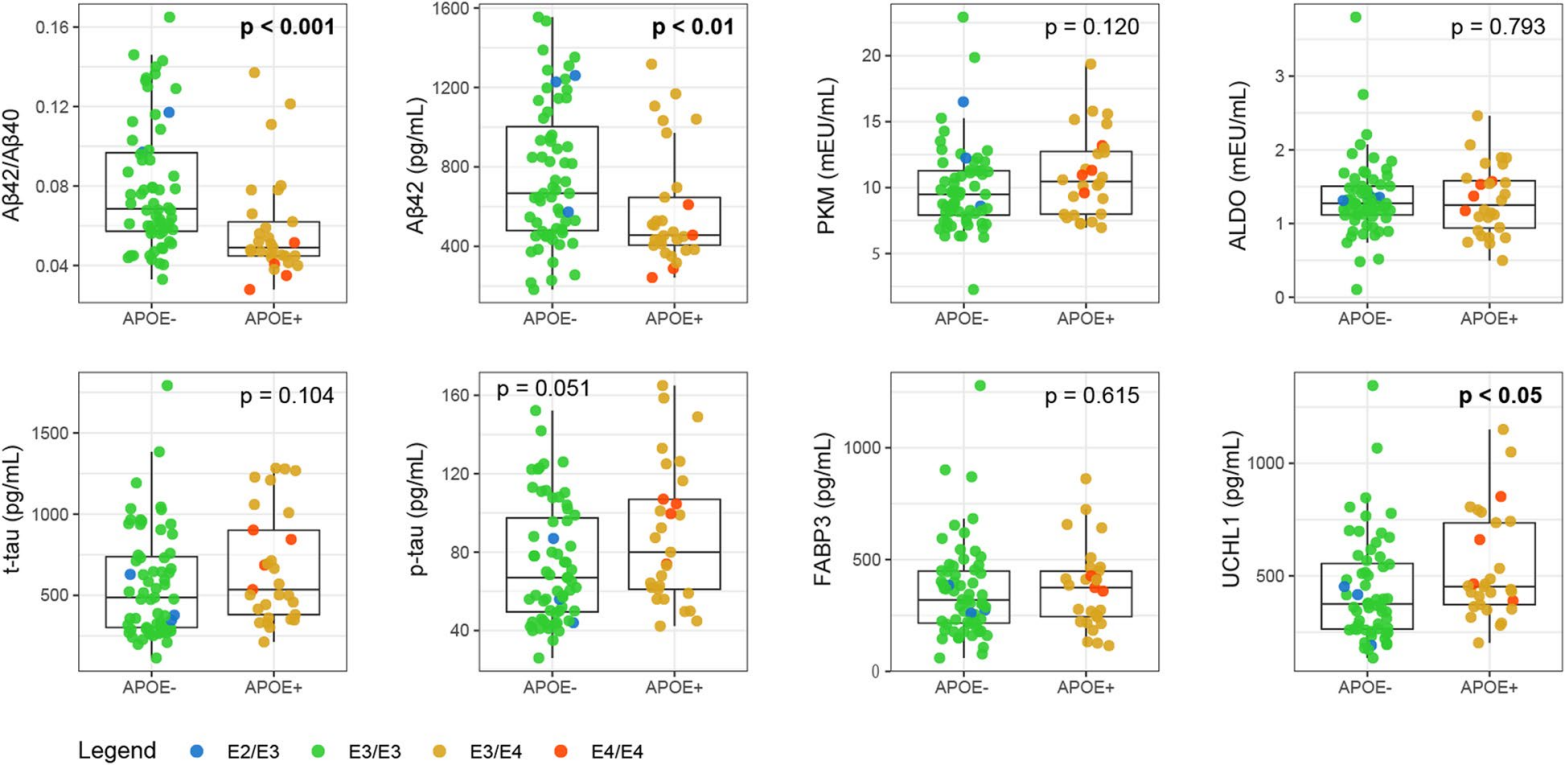
Level of biomarkers according to *APOE* genotype. Combined boxplots and strip plots showing the CSF activity of PKM (mEU/mL) and ALDO (mEU/mL) and the concentration of FABP3 (pg/mL) and UCHL1 (pg/mL) in *APOE*—(ε2/ε3 or ε3/ε3) and *APOE* + (ε4/ε4 or ε3/ε4) patients, independently from the clinical diagnosis. Data are shown as median and interquartile range

We also investigated the effects of *APOE* genotype on the levels and activity of the CSF biomarkers on a subgroup of 93 patients for whom this information was available (AD, *n* = 18; MCI-AD, *n* = 30; pre-AD, *n* = 9; MCI, *n* = 12; CTRL, *n* = 17; FTD, *n* = 7) (Fig. 3).

The presence of at least one *APOE* ε4 allele had a clear influence on the levels of the amyloid peptides. Indeed, significantly lower levels of the Aβ42/40 ratio (*p* < 0.001) in CSF of patients with *APOE* ε4 genotype (*APOE* + : ε4/ε4 or ε3/ε4) were observed compared to those carrying the *APOE*—genotype (ε2/ε3 or ε3/ε3). The same trend was observed for Aβ42 alone (*p* < 0.01). Conversely, higher, though not significant, t-tau and p-tau levels were found in *APOE* + patients.

Among the new candidate biomarkers, UCHL1 showed significantly higher levels in *APOE* + patients (*p* < 0.05), whereas similar activity/concentrations were observed for PKM, ALDO, and FABP3 across the different genotypes.

## Discussion

In this work we investigated the potential role of PKM, ALDO, FABP3, and UCHL1 as biomarkers to characterize AD patients across the disease continuum, with particular interest for the preclinical and early stages of the disease.

Our data showed increased levels of UCHL1 and FABP3 in AD patients compared to non-AD (i.e., SCD, MCI, and FTD). The two markers had a good diagnostic performance across the AD spectrum, with UCHL1 showing the best capability to differentiate early AD (both preclinical and mild cognitive impairment) from SCD and MCI subjects. Higher levels of UCHL1 were previously found in CSF from a small group of AD patients compared with healthy controls, stable MCI, and patients affected by other dementias [35]. UCHL1 is an enzyme particularly abundant in brain, expressed mostly in neurons [36]. This protein is involved in the regulation of the UPS, one of the main pathways of the cellular proteostasis network [37, 38]. UCHL1 removes ubiquitin from proteins destined to the proteasome pathway (e.g., excess, oxidized or misfolded proteins), both in physiological and pathological conditions [37]. Impaired UCHL1 activity has been associated with several neurodegenerative diseases including AD, Parkinson’s disease, and Amyotrophic Lateral Sclerosis [35, 39–41]. Increased CSF levels of UCHL1 have been previously linked to neuronal loss and traumatic brain injury [42, 43]; this association with neurodegeneration is supported by the strong positive correlation we found between UCHL1 and tau markers in our cohort. On the other hand, UCHL1 levels were similar between patients affected by FTD and control groups. This result is in line with the data reported by Barschke and co-workers, who found no difference for UCHL1 levels in CSF of FTD patients carrying mutations in the C9orf72 gene with respect to non-carrier control subjects [44].

We also found significantly higher UCHL1 levels in CSF of patients with at least one *APOE* ε4 allele. To the best of our knowledge, this result has never been reported before, and it would be interesting to further investigate this association in larger cohorts.

Also FABP3 showed good diagnostic performance in discriminating AD patients from controls, with increased median levels of FABP3 already at the pre-AD stage. This confirms FABP3 as a sensitive biomarker of pre-dementia neurodegeneration as previously reported [30, 32]. However, FABP3 levels were similar between FTD and controls, with a similar pattern to UCHL1 across the AD spectrum. Previous studies showed a significant increase of FABP3 in CSF of AD patients [30, 32, 45, 46], as well as in other neurodegenerative disorders, including Creutzfeldt-Jacob disease (CJD), vascular dementia (VAD), and Dementia with Lewy Bodies (DLB) [32, 47, 48], while no data were available for FTD patients before our work. In summary, FABP3 shows a different behaviour across neurodegenerative disorders. The reason of these differences is currently unknown, and further cross-sectional studies should be undertaken to better understand the specificity of this marker for AD and its involvement in the pathological processes leading to dementia.

We also measured the activity of the glycolytic enzymes PKM and ALDO in the selected cohort. The activity of these two enzymes was previously assayed post-mortem in brain tissues of AD patients and controls, revealing a significant increase of PKM activity in AD [49]. Our work represents the first study in which CSF PKM and ALDO activities were measured in a large and well-characterized cohort across the AD continuum. While ALDO did not show any difference among the diagnostic groups, PKM activity was increased in AD patients already at the preclinical stage. Interestingly, PKM activity was increased also in FTD patients, showing levels similar to the ADdem group. Both FTD and AD are characterised by glucose hypometabolism in the brain, usually assessed with FDG-PET to support differential diagnosis since the areas interested during disease progression are different [50, 51]. The increase of CSF PKM activity in both AD and FTD patients might be assumed as a proxy biomarker either for altered glucose metabolism occurring in the brain of these subjects [52] or for a general neurodegeneration process, where PKM is released in CSF due to cell death. Importantly, the change in PKM activity in pre-AD patients may support the view that alteration of glucose metabolism could be an early event even preceding the onset of clinical symptoms as it has been found using imaging techniques [53]. Mechanistic evidence also supports the impact of glucose metabolism alterations in AD pathogenesis. PKM catalyses the transfer of a phosphoryl group from phosphoenolpyruvate (PEP) to ADP generating ATP and pyruvate in a rate-limiting step of glycolysis. Pyruvate can be further metabolized in mitochondria to produce ATP via oxidative phosphorylation or fermented in the cytosol by lactate dehydrogenase (LDH) to produce lactic acid. However, in some conditions, lactate is produced despite the presence of oxygen, switching ATP production from oxidative phosphorylation to aerobic glycolysis in a process described by Warburg [54]. This effect has been studied also in AD, where it seems to induce a de-differentiation process, linked to several deficits which are considered hallmark of AD like synaptic failure, neuronal degeneration, and activation of apoptotic pathways [54–56]. A recent work showed that PKM is involved in this metabolic reprogramming towards aerobic glycolysis and acts not just as an enzyme of glucose catabolism, but also as a nuclear factor promoting neuronal fate loss and vulnerability [54]. PKM is strongly expressed in brain of AD patients and seems to regulate γ-secretase activity and, indirectly, the production of the Aβ peptides [55]. Of interest, it has been shown that the stability of PKM is regulated by the deubiquitinating activity of UCHL1 [56], suggesting an indirect link between UCHL1 and glucose metabolism.

When interpreting our results, it should be noted that they are limited by a relatively small size of the pre-AD group. Additionally, our enzyme assay for PKM measures the total kinase activity of all isoforms which may be present in brain and CSF with different functional roles [57]. Therefore, the use of complementary techniques (i.e., ELISA or mass spectrometry) is warranted to allow the identification and quantification of PKM isoforms in CSF, possibly clarifying the specific roles of these proteins in neurodegeneration processes. Considering the interesting results we obtained for this enzyme, detailed pre-analytical assessment of PKM activity in CSF should also be carried out to define operating procedures for the measurement of this enzyme in CSF. Finally, considering the evidence for a dysfunctional carbohydrate metabolism in AD [52], the characterization and measurement of other key glycolytic enzymes is needed to fully understand how metabolic changes impact AD pathogenesis.

## Conclusions

In conclusion, our study confirms the potential role of UCHL1 and FABP3 as biomarkers of AD neurodegenerative processes. Furthermore, our results validated PKM activity as a novel CSF biomarker to monitor changes of the glycolytic pathway in AD and FTD. The alteration of these proteins in CSF allowed to recognize AD patients already at the preclinical stage of the disease and before cognitive impairment occurred. However, while UCHL1 and FABP3 appeared to be specific for AD, PKM activity increased in both AD and FTD patients, underlining that these two diseases may share alterations of energy metabolism that should be further explored as candidate pathways involved in dementia.

## Supplementary Information

**Additional file 1.**

## Data Availability

Datasets of the study are available from the corresponding authors upon request.

## Abbreviations

AD: Alzheimer’s disease
ADdem: AD with dementia
ALDO: Fructose-bisphosphate aldolase
AT(N): Amyloidosis, Tauopathy and Neurodegeneration biomarkers
Aβ peptide: Amyloid-β peptide
CDR: Clinical dementia rating
CJD: Creutzfeldt-Jacob disease
CSF: Cerebrospinal fluid
DLB: Dementia with Lewy Bodies
FABP3: Fatty acid-binding protein 3
FDG-PET: 18Fluoro-2-deoxyglucose positron emission tomography
FTD: Frontotemporal dementia
LDH: Lactate dehydrogenase
MCI: Mild cognitive impairment
MMSE: Mini-Mental State Examination
MRI: Magnetic resonance imaging
NIA-AA: National Institute on Aging—Alzheimer's Association
ON: Overnight
PDD: Parkinson’s disease with Dementia
PEP: Phosphoenolpyruvate
PKM: Pyruvate kinase isoform M
pre-AD: Preclinical AD
p-tau: Hyperphosphorylated tau protein
ROC: Receiver Operator Characteristics analysis
RT: Room temperature
SCD: Subjective cognitive decline
SEM: Standard error of the mean
t-tau: Total tau
UCHL1: Ubiquitin C-terminal hydrolase L1
UPS: Proteasomal system
VAD: Vascular dementia

## References

[1] Jack CR, Bennett DA, Blennow K, Carrillo MC, Dunn B, Haeberlein SB, et al. NIA-AA Research Framework: Toward a biological definition of Alzheimer’s disease. Alzheimers Dement 2018;14(4):535–62. 10.1016/j.jalz.2018.02.018 PMC5958625 29653606

[2] Therriault J, Zimmer ER, Benedet AL, Pascoal TA, Gauthier S, Rosa-Neto P (2022). Staging of Alzheimer’s disease: past, present, and future perspectives. Trends Mol Med.

[3] Blennow K, de Leon MJ, Zetterberg H (2006). Alzheimer’s disease. Lancet.

[4] Jack CR, Bennett DA, Blennow K, Carrillo MC, Feldman HH, Frisoni GB (2016). A/T/N: An unbiased descriptive classification scheme for Alzheimer disease biomarkers. Neurology.

[5] Long JM, Coble DW, Xiong C, Schindler SE, Perrin RJ, Gordon BA (2022). Preclinical Alzheimer’s disease biomarkers accurately predict cognitive and neuropathological outcomes. Brain.

[6] Calabrò M, Rinaldi C, Santoro G, Crisafulli C (2021). The biological pathways of Alzheimer disease: a review. AIMS Neurosci.

[7] Hardy J, Bogdanovic N, Winblad B, Portelius E, Andreasen N, Cedazo-Minguez A (2014). Pathways to Alzheimer’s disease. J Intern Med.

[8] Johnson ECB, Dammer EB, Duong DM, Ping L, Zhou M, Yin L (2020). Large-scale proteomic analysis of Alzheimer’s disease brain and cerebrospinal fluid reveals early changes in energy metabolism associated with microglia and astrocyte activation. Nat Med.

[9] Higginbotham L, Ping L, Dammer EB, Duong DM, Zhou M, Gearing M (2020). Integrated proteomics reveals brain-based cerebrospinal fluid biomarkers in asymptomatic and symptomatic Alzheimer’s disease. Sci Adv.

[10] Zhou M, Haque RU, Dammer EB, Duong DM, Ping L, Johnson ECB (2020). Targeted mass spectrometry to quantify brain-derived cerebrospinal fluid biomarkers in Alzheimer’s disease. Clin Proteomics.

[11] Bader JM, Geyer PE, Müller JB, Strauss MT, Koch M, Leypoldt F (2020). Proteome profiling in cerebrospinal fluid reveals novel biomarkers of Alzheimer’s disease. Mol Syst Biol.

[12] Castrillo JI, Lista S, Hampel H, Ritchie CW (2018). Systems biology methods for Alzheimer’s disease research toward molecular signatures, subtypes, and stages and precision medicine: Application in cohort studies and trials. Methods Mol Biol.

[13] van Zalm PW, Ahmed S, Fatou B, Schreiber R, Barnaby O, Boxer A (2023). Meta-analysis of published cerebrospinal fluid proteomics data identifies and validates metabolic enzyme panel as Alzheimer’s disease biomarkers. Cell Rep Med.

[14] Lowe J, McDermott H, Landon M, Mayer RJ, Wilkinson KD (1990). Ubiquitin carboxyl-terminal hydrolase (PGP 9.5) is selectively present in ubiquitinated inclusion bodies characteristic of human neurodegenerative diseases. J Pathol.

[15] Furuhashi M, Hotamisligil GS (2008). Fatty acid-binding proteins: Role in metabolic diseases and potential as drug targets. Nat Rev Drug Discov.

[16] del Campo M, Peeters CFW, Johnson ECB, Vermunt L, Hok-A-Hin YS, van Nee M (2022). CSF proteome profiling across the Alzheimer’s disease spectrum reflects the multifactorial nature of the disease and identifies specific biomarker panels. Nat Aging.

[17] Bellomo G, Indaco A, Chiasserini D, Maderna E, Paolini Paoletti F, Gaetani L (2021). Machine Learning Driven Profiling of Cerebrospinal Fluid Core Biomarkers in Alzheimer’s Disease and Other Neurological Disorders. Front Neurosci.

[18] Albert MS, DeKosky ST, Dickson D, Dubois B, Feldman HH, Fox NC (2011). The diagnosis of mild cognitive impairment due to Alzheimer’s disease: Recommendations from the National Institute on Aging-Alzheimer’s Association workgroups on diagnostic guidelines for Alzheimer’s disease. Alzheimers Dement.

[19] Rascovsky K, Hodges JR, Knopman D, Mendez MF, Kramer JH, Neuhaus J (2011). Sensitivity of revised diagnostic criteria for the behavioural variant of frontotemporal dementia. Brain.

[20] Gorno-Tempini ML, Hillis AE, Weintraub S, Kertesz A, Mendez M, Cappa SF (2011). Classification of primary progressive aphasia and its variants. Neurology.

[21] Jessen F, Amariglio RE, Van Boxtel M, Breteler M, Ceccaldi M, Chételat G (2014). A conceptual framework for research on subjective cognitive decline in preclinical Alzheimer’s disease. Alzheimers Dement.

[22] Hansson O, Batrla R, Brix B, Carrillo MC, Corradini V, Edelmayer RM (2021). The Alzheimer’s Association international guidelines for handling of cerebrospinal fluid for routine clinical measurements of amyloid β and tau. Alzheimers Dement.

[23] Del Campo M, Mollenhauer B, Bertolotto A, Engelborghs S, Hampel H, Simonsen AH (2012). Recommendations to standardize preanalytical confounding factors in Alzheimers and Parkinsons disease cerebrospinal fluid biomarkers: An update. Biomark Med.

[24] Teunissen CE, Tumani H, Bennett JL, Berven FS, Brundin L, Comabella M (2011). Consensus Guidelines for CSF and Blood Biobanking for CNS Biomarker Studies. Mult Scler Int.

[25] Chiasserini D, Biscetti L, Farotti L, Eusebi P, Salvadori N, Lisetti V (2016). Performance evaluation of an automated ELISA system for Alzheimer’s disease detection in clinical routine. Journal of Alzheimer’s Disease.

[26] Gutmann I, Bernt E. Pyruvate Kinase Assay in Serum and Erythrocytes. Methods of Enzymatic Analysis. 2nd ed. Elsevier; 1974.

[27] Dawson NJ, Biggar KK, Storey KB (2013). Characterization of Fructose-1,6-Bisphosphate Aldolase during Anoxia in the Tolerant Turtle, Trachemys scripta elegans: An Assessment of Enzyme Activity. Expression and Structure. PLoS One..

[28] Tsutsumi H, Tani K, Fujii H, Miwa S (1988). Expression of L- and M-type pyruvate kinase in human tissues. Genomics.

[29] Chang YC, Yang YC, Tien CP, Yang CJ, Hsiao M (2018). Roles of Aldolase Family Genes in Human Cancers and Diseases. Trends Endocrinol Metab.

[30] Chiasserini D, Parnetti L, Andreasson U, Zetterberg H, Giannandrea D, Calabresi P (2010). CSF Levels of Heart Fatty Acid Binding Protein are Altered During Early Phases of Alzheimer’s Disease. J Alzheimers Dis.

[31] R Core Team 2019. R: A language and environment for statistical computing. R Foundation for Statistical Computing, Vienna, Austria. URL http://www.R-project.org/. 2019.

[32] Chiasserini D, Biscetti L, Eusebi P, Salvadori N, Frattini G, Simoni S (2017). Differential role of CSF fatty acid binding protein 3, α-synuclein, and Alzheimer’s disease core biomarkers in Lewy body disorders and Alzheimer’s dementia. Alzheimers Res Ther.

[33] Robin X, Turck N, Hainard A, Tiberti N, Lisacek F, Sanchez JC (2011). pROC: An open-source package for R and S+ to analyze and compare ROC curves. BMC Bioinformatics.

[34] Venables WN, Ripley BD (2002). Modern Applied Statistics with S.

[35] Öhrfelt A, Johansson P, Wallin A, Andreasson U, Zetterberg H, Blennow K (2017). Increased Cerebrospinal Fluid Levels of Ubiquitin Carboxyl-Terminal Hydrolase L1 in Patients with Alzheimer’s Disease. Dement Geriatr Cogn Dis.

[36] Doran JF, Jackson P, Kynoch PAM, Thompson RJ (1983). Isolation of PGP 9.5, a New Human Neurone-Specific Protein Detected by High-Resolution Two-Dimensional Electrophoresis. J Neurochem.

[37] Osaka H, Wang YL, Takada K, Takizawa S, Setsuie R, Li H (2003). Ubiquitin carboxy-terminal hydrolase L1 binds to and stabilizes monoubiquitin in neuron. Hum Mol Genet.

[38] Liu Y, Fallon L, Lashuel HA, Liu Z, Lansbury PT (2002). The UCH-L1 gene encodes two opposing enzymatic activities that affect α-synuclein degradation and Parkinson’s disease susceptibility. Cell.

[39] Choi J, Levey AI, Weintraub ST, Rees HD, Gearing M, Chin LS (2004). Oxidative modifications and down-regulation of ubiquitin carboxyl-terminal hydrolase L1 associated with idiopathic Parkinson’s and Alzheimer’s diseases. J Biol Chem.

[40] Zhang M, Cai F, Zhang S, Zhang S, Song W (2014). Overexpression of ubiquitin carboxyl-terminal hydrolase L1 (UCHL1) delays Alzheimer’s progression in vivo. Sci Rep.

[41] Li R, Wang J, Xie W, Liu J, Wang C (2020). UCHL1 from serum and CSF is a candidate biomarker for amyotrophic lateral sclerosis. Ann Clin Transl Neurol.

[42] Stukas S, Gill J, Cooper J, Belanger L, Ritchie L, Tsang A (2021). Characterization of cerebrospinal fluid ubiquitin C-Terminal hydrolase L1 as a biomarker of human acute traumatic spinal cord injury. J Neurotrauma.

[43] Gorgoraptis N, Li LM, Whittington A, Zimmerman KA, Maclean LM, McLeod C (2019). In vivo detection of cerebral tau pathology in long-term survivors of traumatic brain injury. Sci Transl Med.

[44] Barschke P, Oeckl P, Steinacker P, Al Shweiki MHDR, Weishaupt JH, Landwehrmeyer GB (2020). Different CSF protein profiles in amyotrophic lateral sclerosis and frontotemporal dementia with C9orf72 hexanucleotide repeat expansion. J Neurol Neurosurg Psychiatry.

[45] Desikan RS, Thompson WK, Holland D, Hess CP, Brewer JB, Zetterberg H (2013). Heart fatty acid binding protein and Aβ-associated Alzheimer’s neurodegeneration. Mol Neurodegener.

[46] Olsson B, Lautner R, Andreasson U, Öhrfelt A, Portelius E, Bjerke M (2016). CSF and blood biomarkers for the diagnosis of Alzheimer’s disease: A systematic review and meta-analysis. Lancet Neurol.

[47] Steinacker P, Mollenhauer B, Bibl M, Cepek L, Esselmann H, Brechlin P (2004). Heart fatty acid binding protein as a potential diagnostic marker for neurodegenerative diseases. Neurosci Lett.

[48] Bjerke M, Kern S, Blennow K, Zetterberg H, Waern M, Börjesson-Hanson A (2015). Cerebrospinal Fluid Fatty Acid-Binding Protein 3 is Related to Dementia Development in a Population-Based Sample of Older Adult Women Followed for 8 Years. J Alzheimers Dis.

[49] Bigl M, Brückner MK, Arendt T, Bigl V, Eschrich K (1999). Activities of key glycolytic enzymes in the brains of patients with Alzheimer’s disease. J Neural Transm.

[50] Chételat G, Arbizu J, Barthel H, Garibotto V, Law I, Morbelli S (2020). Amyloid-PET and 18F-FDG-PET in the diagnostic investigation of Alzheimer’s disease and other dementias. Lancet Neurol.

[51] Minoshima S, Cross D, Thientunyakit T, Foster NL, Drzezga A (2022). (18)F-FDG PET Imaging in Neurodegenerative Dementing Disorders: Insights into Subtype Classification, Emerging Disease Categories, and Mixed Dementia with Copathologies. J Nucl Med.

[52] Panyard DJ, McKetney J, Deming YK, Morrow AR, Ennis GE, Jonaitis EM, et al. Large-scale proteome and metabolome analysis of CSF implicates altered glucose and carbon metabolism and succinylcarnitine in Alzheimer's disease. Alzheimers Dement 2023; 10.1002/alz.13130. 10.1002/alz.13130 PMC10663389 37218097

[53] Ou YN, Xu W, Li JQ, Guo Y, Cui M, Chen KL (2019). FDG-PET as an independent biomarker for Alzheimer’s biological diagnosis: A longitudinal study. Alzheimers Res Ther.

[54] Traxler L, Herdy JR, Stefanoni D, Eichhorner S, Pelucchi S, Szücs A (2022). Warburg-like metabolic transformation underlies neuronal degeneration in sporadic Alzheimer’s disease. Cell Metab.

[55] Han J, Hyun J, Park J, Jung S, Oh Y, Kim Y (2021). Aberrant role of pyruvate kinase M2 in the regulation of gamma-secretase and memory deficits in Alzheimer’s disease. Cell Rep.

[56] Ham SJ, Lee D, Xu WJ, Cho E, Choi S, Min S (2021). Loss of UCHL1 rescues the defects related to Parkinson’s disease by suppressing glycolysis. Sci Adv.

[57] Zhang Z, Deng X, Liu Y, Liu Y, Sun L, Chen F. PKM2, function and expression and regulation. 2019; Cell Biosci. 2019;9:52. 10.1186/s13578-019-0317-8 PMC6595688 31391918

