# Supplementary material for: Potential diagnostic value of CSF metabolism-related proteins across the Alzheimer’s disease continuum
Source: Alzheimers Res Ther. 2023 Jul 15;15:124. doi: 10.1186/s13195-023-01269-8 (PMC10350263; doi:10.1186/s13195-023-01269-8)
Supplement: Supplementary file 1 — Additional file 1. [file 13195_2023_1269_MOESM1_ESM.docx]

**SUPPLEMENTARY MATERIALS**

**Supplementary table 1.** Coefficient of variation for the biomarker assays used in this work

| **Biomarker** | **Intra-assay CV (%)** | **Inter-assay CV (%)** |
| --- | --- | --- |
| PKM | 4.3 | 6.7 |
| ALDO | 3.7 | 6.3 |
| FABP3 | 3.4 | 9.7 |
| UCHL1 | 2.9 | 3.0 |

**Supplementary Table 2.** Analysis of covariance between the diagnostic groups

| **Comparisons** | **PKM** | **ALDO** | **UCHL1** | **FABP3** | **MMSE** |
| --- | --- | --- | --- | --- | --- |
| **SCD vs. ADdem** | **0.007** | 0.924 | **<0.001** | **<0.001** | **<0.001** |
| **FTD vs. ADdem** | 0.957 | 1.000 | **0.006** | **0.028** | **<0.001** |
| **MCI vs. ADdem** | **<0.001** | 0.096 | **<0.001** | **<0.001** | **<0.001** |
| **MCI-AD vs. ADdem** | 1.000 | 0.998 | 0.996 | 0.999 | **<0.001** |
| **pre-AD vs. ADdem** | 0.969 | 0.992 | 1.000 | 0.925 | **<0.001** |
| **FTD vs. SCD** | **<0.001** | 0.951 | 0.372 | 0.565 | **<0.001** |
| **MCI vs. SCD** | 0.605 | 0.514 | 1.000 | 0.988 | **<0.001** |
| **MCI-AD vs. SCD** | **0.004** | 0.992 | **<0.001** | **<0.001** | **<0.001** |
| **pre-AD vs. SCD** | 0.377 | 1.000 | **0.001** | **0.047** | 0.972 |
| **MCI vs. FTD** | **<0.001** | 0.163 | 0.466 | 0.315 | 0.758 |
| **MCI-AD vs. FTD** | 0.975 | 1.000 | **0.024** | **0.010** | 1.000 |
| **pre-AD vs. FTD** | 0.720 | 0.997 | 0.129 | 0.623 | **<0.001** |
| **MCI-AD vs. MCI** | **<0.001** | 0.216 | **<0.001** | **<0.001** | 0.683 |
| **pre-AD vs. MCI** | **0.023** | 0.616 | **0.001** | **0.013** | 0.006 |
| **pre-AD vs. MCI-AD** | 0.952 | 1.000 | 1.000 | 0.808 | < 0.001 |

The table reports the p-values obtained in the analysis of covariance (ANCOVA), adjusted for multiple comparisons. Bold font indicates the statistical significance threshold (p<0.05). ADdem: Alzheimer’s disease with dementia, FTD: frontotemporal dementia; MCI: mild cognitive impairment, MCI-AD: mild cognitive impairment due to AD, pre-AD: preclinical AD, SCD: subjective cognitive decline; MMSE Mini Mental State Examination

***Supplementary Table 2*** *Biomarker levels in CSF*

| **Variable** | **Overall** | **SCD** | **MCI** | **pre-AD** | **MCI-AD** | **ADdem** | **FTD** |
| --- | --- | --- | --- | --- | --- | --- | --- |
| **n** | 233 | 52 | 30 | 19 | 50 | 45 | 37 |
| **Aβ42**  median [IQR] | 695  [498, 1103] | 1084  [738, 1358] | 1072  [744, 1255] | 648  [572, 815] | 523  [435, 712] | 506  [405, 573] | 956  [648, 1180] |
| **Aβ42/40**  median [IQR] | 0.068  [0.05, 0.10] | 0.100  [0.09, 0.12] | 0.101  [0.08, 0.12] | 0.054  [0.04, 0.06] | 0.054  [0.04, 0.06] | 0.048  [0.04, 0.06] | 0.100  [0.08, 0.13] |
| **p-Tau (pg/mL)**  median [IQR] | 60.00  [42.30, 91.80] | 41.50  [30.27, 49.00] | 42.90  [34.50, 49.42] | 73.20  [60.50, 96.70] | 91.10  [67.25, 111] | 102  [72.00, 122] | 43.70  [33.40, 52.90] |
| **t-Tau (pg/mL)**  median [IQR] | 407  [272, 695] | 267  [199, 336] | 283  [225, 334] | 470  [377, 681] | 618  [503, 833] | 875  [594, 1028] | 304  [232, 355] |
| **PKM (mEU/mL)**  median [IQR] | 9.41  [7.60, 11.35] | 8.22  [6.64, 9.61] | 7.40  [6.67, 9.21] | 10.60  [8.01, 13.95] | 9.86  [8.59, 12.25] | 10.24  [8.78, 12.07] | 10.02  [8.96, 12.90] |
| **ALDO (mEU/mL)**  median [IQR] | 1.18  [0.89, 1.51] | 1.10  [0.85, 1.40] | 1.08  [0.78, 1.28] | 1.31  [0.90, 1.51] | 1.31  [1.04, 1.55] | 1.28  [1.07, 1.55] | 1.11  [0.80, 1.61] |
| **FABP3 (pg/mL)**  median [IQR] | 289  [195, 414] | 197  [150, 307] | 190  [147, 273] | 323  [245, 418] | 395  [286, 532] | 362  [279, 473] | 255  [181, 332] |
| **UCHL1 (pg/mL)**  median [IQR] | 393  [282, 551] | 268  [220, 391] | 273  [207, 365] | 454  [369, 776] | 478  [372, 669] | 540  [394, 728] | 374  [261, 441] |

ADdem : Alzheimer’s disease with dementia; FTD: frontotemporal dementia; IQR: interquartile range; MCI: mild cognitive impairment; MCI-AD: mild cognitive impairment due to AD, pre-AD: preclinical AD, SCD: subjective cognitive decline

***Supplementary Table 3*** Correlation matrix (Spearman) between CSF biomarkers and demographics and neuropsychological parameters in the whole cohort

|  | Age at LP | MMSE_T0 | ΔMMSE_1.5y |
| --- | --- | --- | --- |
| Aβ42 | -0.13  n.s. | 0.32  ***  ♦ | 0.32  * |
| Aβ42/40 ratio | -0.40  ***  ♦ | 0.30  ***  ♦ | 0.17  n.s |
| t-Tau | 0.28  ***  ♦ | -0.41  ***  ♦ | -0.20  n.s |
| p-Tau | 0.27  ***  ♦ | -0.33  ***  ♦ | -0.13  n.s |
| PKM | 0.29  ***  ♦ | -0.13  n.s. | 0.06  n.s |
| ALDO | 0.26  ***  ♦ | -0.08  n.s. | 0.09  n.s |
| FABP3 | 0.29  ***  ♦ | -0.16  * | -0.02  n.s |
| UCHL1 | 0.24  **  ♦ | -0.27  ***  ♦ | -0.19  n.s |

Asterisks indicate level of statistical significance: *p≤0.05, **p≤0.001, ***p≤0.0001; n.s.: not significant. ♦ indicates the significant Spearman correlations adjusted for multiple comparisons according to the Bonferroni correction method (p ≤ 0.00038). LP= lumbar puncture.

**Supplementary Table 4** Receiver operator characteristic (ROC) analysis of the new candidate CSF biomarkers between the diagnostic groups

| Group comparisons | Biomarker | AUC (95% CI) | Specificity | Sensitivity |
| --- | --- | --- | --- | --- |
| SCD vs. ADdem | UCHL1 | 0.84 (0.77-0.92) | 0.67 | 0.89 |
|  | FABP3 | 0.81 (0.72-0.90) | 0.71 | 0.89 |
|  | PKM | 0.77 (0.67-0.86) | 0.60 | 0.82 |
| SCD vs. MCI-AD | UCHL1 | 0.83 (0.75-0.91) | 0.67 | 0.88 |
|  | FABP3 | 0.83 (0.75-0.91) | 0.63 | 0.92 |
|  | PKM | 0.77 (0.67-0.86) | 0.60 | 0.82 |
| SCD vs. PRE-AD | UCHL1 | 0.83 (0.74-0.93) | 0.67 | 0.94 |
|  | FABP3 | 0.78 (0.67-0.89) | 0.47 | 1.00 |
|  | PKM | 0.69 (0.53-0.86) | 0.88 | 0.56 |
| MCI vs. ADdem | UCHL1 | 0.82 (0.71-0.93) | 0.73 | 0.89 |
|  | FABP3 | 0.81 (0.70-0.92) | 0.73 | 0.82 |
|  | PKM | 0.78 (0.67-0.89) | 0.70 | 0.86 |
| MCI vs. MCI-AD | UCHL1 | 0.80 (0.69-0.92) | 0.73 | 0.88 |
|  | FABP3 | 0.82 (0.72-0.92) | 0.57 | 0.96 |
|  | PKM | 0.80 (0.70-0.91) | 0.70 | 0.88 |
| MCI vs. PRE-AD | UCHL1 | 0.82 (0.70-0.94) | 0.67 | 0.94 |
|  | FABP3 | 0.77 (0.64-0.90) | 0.57 | 0.89 |
|  | PKM | 0.73 (0.57-0.89) | 0.70 | 0.78 |
| CTRL vs. AD continuum | UCHL1 | 0.83 (0.77-0.89) | 0.69 | 0.88 |
|  | FABP3 | 0.81 (0.75-0.87) | 0.57 | 0.95 |
|  | PKM | 0.77 (0.70-0.83) | 0.55 | 0.86 |
| CTRL vs. ADdem | UCHL1 | 0.83 (0.76-0.91) | 0.69 | 0.89 |
|  | FABP3 | 0.81 (0.73-0.89) | 0.68 | 0.89 |
|  | PKM | 0.77 (0.69-0.86) | 0.64 | 0.82 |
| CTRL vs. MCI-AD | UCHL1 | 0.82 (0.75-0.89) | 0.69 | 0.88 |
|  | FABP3 | 0.82 (0.75-0.89) | 0.62 | 0.92 |
|  | PKM | 0.78 (0.70-0.86) | 0.56 | 0.88 |
| CTRL vs. PRE-AD | UCHL1 | 0.83 (0.74-0.91) | 0.67 | 0.94 |
|  | FABP3 | 0.77 (0.68-0.87) | 0.57 | 0.89 |
|  | PKM | 0.71 (0.55-0.87) | 0.85 | 0.56 |
| FTD vs. SCD | UCHL1 | 0.64 (0.52-0.76) | 0.67 | 0.65 |
|  | FABP3 | 0.62 (0.50-0.74) | 0.71 | 0.57 |
|  | PKM | 0.77 (0.68-0.87) | 0.60 | 0.86 |
| FTD vs. MCI | UCHL1 | 0.63 (0.49-0.77) | 0.67 | 0.65 |
|  | FABP3 | 0.61 (0.47-0.75) | 0.50 | 0.74 |
|  | PKM | 0.80 (0.69-0.91) | 0.73 | 0.83 |
| FTD vs. CTRL | UCHL1 | 0.64 (0.53-0.75) | 0.67 | 0.65 |
|  | FABP3 | 0.61 (0.50-0.72) | 0.68 | 0.57 |
|  | PKM | 0.78 (0.69-0.87) | 0.64 | 0.86 |
| FTD vs. ADdem | UCHL1 | 0.75 (0.64-0.85) | 0.51 | 0.89 |
|  | FABP3 | 0.73 (0.62-0.84) | 0.82 | 0.54 |
|  | PKM | 0.48 (0.35-0.61) | 0.55 | 0.53 |
| FTD vs. MCI-AD | UCHL1 | 0.73 (0.62-0.83) | 0.58 | 0.78 |
|  | FABP3 | 0.75 (0.64-0.85) | 0.61 | 0.80 |
|  | PKM | 0.52 (0.40-0.65) | 0.33 | 0.81 |
| FTD vs. PRE-AD | UCHL1 | 0.72 (0.58-0.86) | 0.61 | 0.73 |
|  | FABP3 | 0.66 (0.51-0.81) | 0.47 | 0.80 |
|  | PKM | 0.48 (0.29-0.66) | 0.28 | 0.86 |
| FTD vs. AD continuum | UCHL1 | 0.73 (0.64-0.83) | 0.78 | 0.58 |
|  | FABP3 | 0.73 (0.63-0.82) | 0.80 | 0.56 |
|  | PKM | 0.48 (0.37-0.59) | 0.61 | 0.48 |

ADdem: Alzheimer’s disease with dementia; FTD: frontotemporal dementia; MCI: mild cognitive impairment; MCI-AD: mild cognitive impairment due to AD; pre-AD: preclinical AD; SCD: subjective cognitive decline; CTRL: control group (SCD + MCI); AD continuum: pre-AD + MCI-AD + ADdem.

***Supplementary Figure 1*** Effect of long-time storage on the stability of the candidate biomarkers.


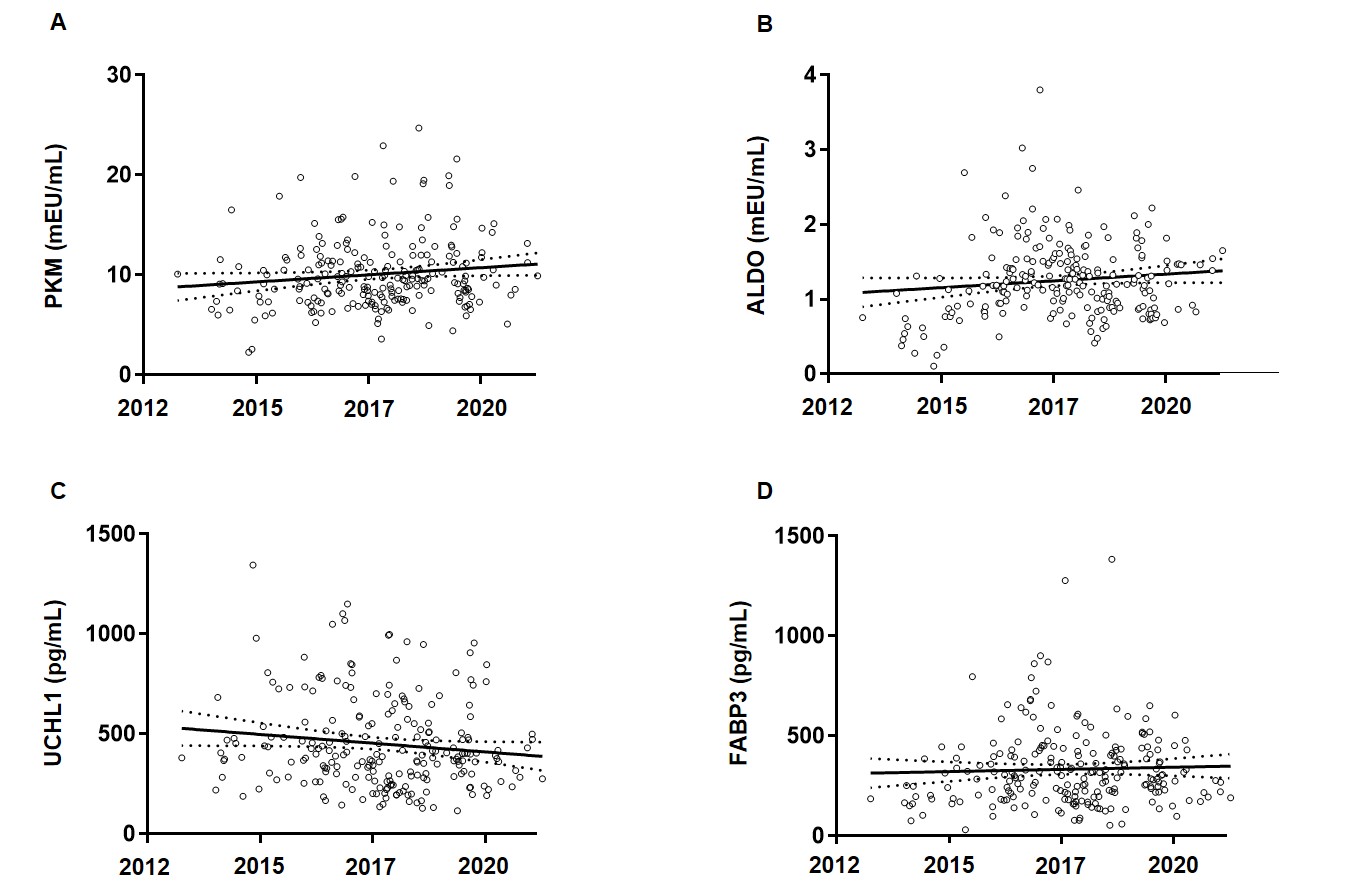


Scatterplots represent the activity of PKM **(A)** and ALDO **(B)** and the concentration of UCHL1 **(C)** and FABP3 **(D)** measured in CSF samples collected between 2013 and 2021. Linear regression analyses showed no significant effects of storage time on biomarker concentrations. Solid lines represent regression fit and dotted lines represent the 95% confidence intervals of the regression models

**Supplementary Figure 2** CSF levels of the core AD biomarkers in the diagnostic groups.


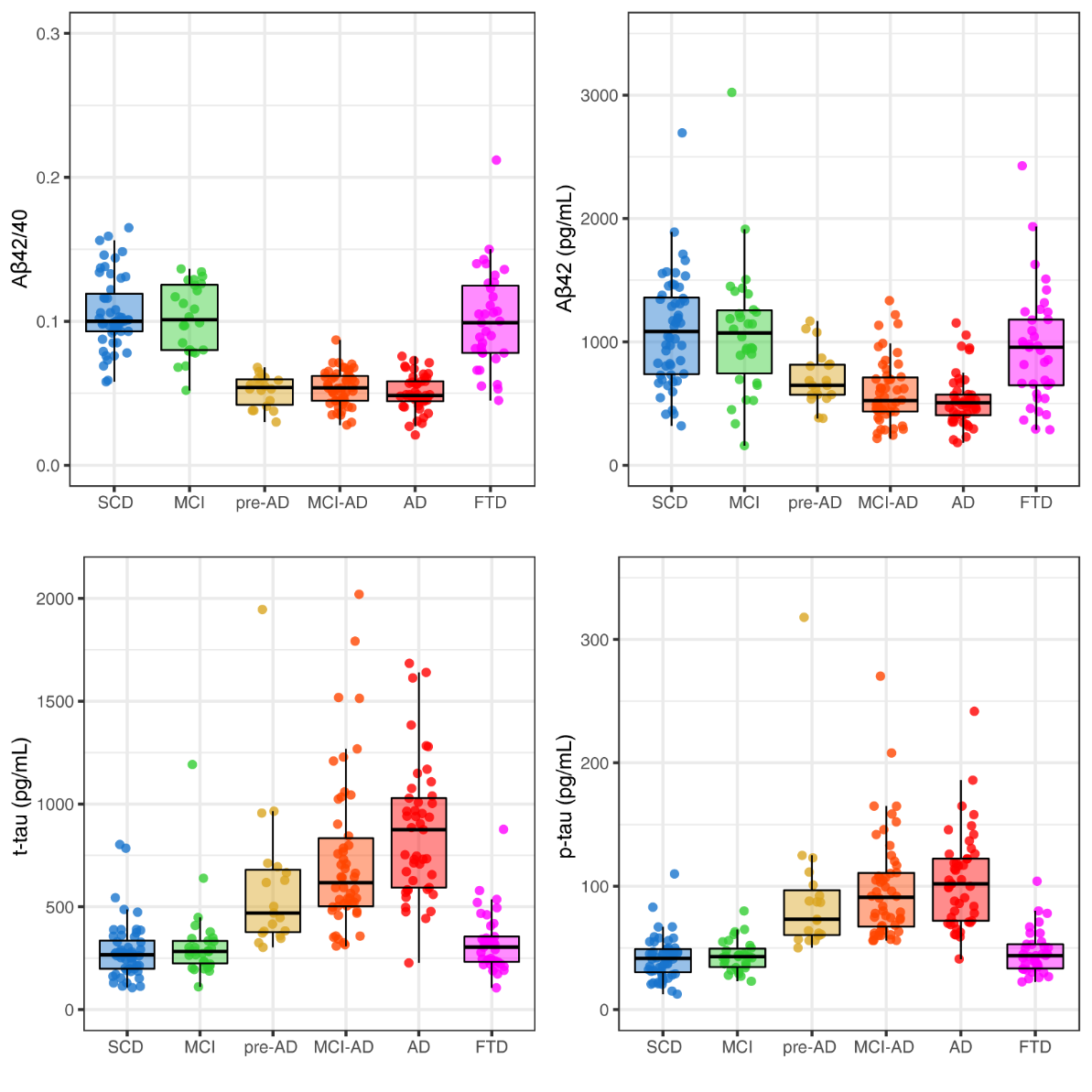


The boxplots show the levels of Aβ42/40 ratio, p-tau, and t-tau in the different diagnostic groups presented as median and interquartile range.
